# Endplates Changes Related to Age and Vertebral Segment

**DOI:** 10.1155/2014/545017

**Published:** 2014-06-12

**Authors:** Carlos Fernando P. S. Herrero, Sergio Britto Garcia, Luis Vicente Garcia, Helton Luiz Aparecido Defino

**Affiliations:** ^1^Department of Biomechanics, Medicine, and Rehabilitation of the Locomotor Apparatus, Faculty of Medicine of Ribeirao Preto, University of Sao Paulo, Bandeirantes Avenue 3900, 14049-900 Ribeirão Preto, SP, Brazil; ^2^Department of Pathology, Faculty of Medicine of Ribeirao Preto, University of Sao Paulo, Bandeirantes Avenue 3900, 14049-900 Ribeirão Preto, SP, Brazil

## Abstract

Endplate separations are defined as the presence of a space between the hyaline cartilage and the cortical bone of the adjacent vertebral body. This study evaluates endplate separations from the vertebral body and intervertebral discs and verifies if endplate separation is related to age and the spinal level. Groups were formed based on age (20–40 and 41–85 years old) and the vertebral segment (T7-T8 and L4-L5 segments). Histological analysis included assessment of the length of the vertebral endplates, the number and dimensions of the separations, and orientation of the collagen fibers, in the mid-sagittal slice. Two indexes were created: the separation index (number of separations/vertebral length) and separation extension index (sum of all separations/vertebral length). The results of the study demonstrated a direct relationship between the density of separations in the endplate and two variables: age and spinal level.

## 1. Introduction

In the adult spine, thin cartilage endplates link the intervertebral discs to the vertebrae. The vertebral endplates possess an osseous and hyaline cartilage layer and were described as a transitional zone between the disc and the adjacent vertebral body in some early anatomical studies [1]. The endplate has important roles in vertebral growth, biomechanical integrity, and disc nutrition [2]. The endplate also has important mechanical functions as a physical barrier to prevent bulging of the nucleus pulposus into the spongiosa of the vertebral body and act as a filter between the disc and vertebral body [3].

The vertebral endplate appears to be the structure of the disc that is most susceptible to mechanical failure, according to microscopic observations and finite element analyses [4]. No interconnection between the cartilage endplate and the lamellar subchondral bone collagen has been observed, which suggests there is no direct physical connection between the endplates and the adjacent bone [5, 6]. The adult intervertebral disc is the largest avascular structure in the body, and there are two main routes for solute transport into the nucleus pulposus: through the endplate and through the annulus fibrosus [7]. The vertebral endplate is the main pathway for nutrients to reach the avascular nucleus pulposus by diffusion from the blood supply of the vertebral body [3]. There is strong evidence that a decrease in nutrient supply is associated with disc degeneration [8]. The vertebral endplate also prevents the loss of smaller proteoglycans from the disc [9]. Vertebral endplate permeability and disc metabolite transport decrease during growth and aging [10].

This study was performed in response to observations and reports of endplate separations from the vertebrae and a possible role for endplate separations in disc degeneration [11, 12]. In disc degeneration, the first morphological change to occur is separation of a segment of the cartilaginous endplate from the adjacent vertebral body; this degenerative process of the cartilaginous endplate advances after the second decade of life [13, 14]. The hypothesis of our study was that endplate separation from the vertebral body would occur more frequently in elderly individuals and in the lumbar spine. The objectives of our study were to evaluate the occurrence of endplate separations from the vertebral body and to verify whether these separations are related to age and vertebral segment.

## 2. Materials and Methods

This study was carried out based on a research protocol approved by the local Committee of Ethics in Research. Thoracic (T7-T8) and lumbar (L4-L5) vertebral segments were harvested during routine autopsy of 41 individuals without any known spinal disease (fracture, tumor, infection, metabolic disease, or previous spinal surgery). Twenty-six cadavers were male (63.4%) and 15 were female (36.5%), and they ranged in age from 20 to 85 years (45.82 ± 17.53 years).

All vertebral segments were removed through an anterior approach. The surrounding tissues were detached from the thoracic (T7-T8) and lumbar (L4-L5) segments; osteotomies were performed at each pedicle level and at the transversal middle of the proximal and distal vertebral bodies of the selected vertebral segments. The discs were examined macroscopically, and radiographs of the vertebral segment were taken in the coronal and sagittal planes. Macroscopic signs of disc degeneration or reduction of the intervertebral disc space, subchondral sclerosis, and the presence of bone cysts and osteophytes on radiographs were parameters used as exclusion criteria for the harvested vertebral segments.

**Figure 1:**
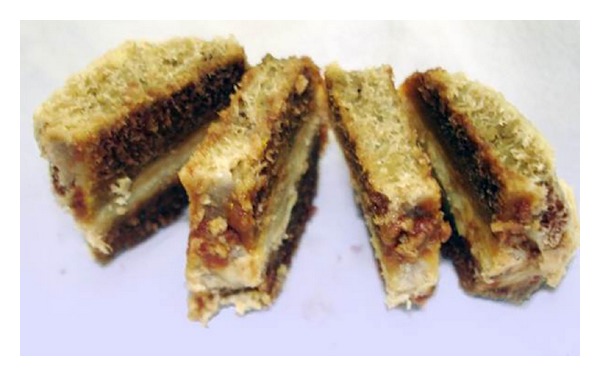
Photographs of front view of the anatomical part of the T7-T8 intervertebral disc and adjacent vertebral bodies after performing the sagittal and of two parasagittal cuts (C).

The thoracic and lumbar segments without macroscopic or radiographic signs of severe degenerative disease were divided at the mid-sagittal plane from bone to intervertebral disc direction. Mid-sagittal slices of each segment, which included parts of the adjacent vertebral bodies, were obtained and processed for histological analyses (Figure 1). The specimens were fixed in 10% neutral formalin and subsequently decalcified in trichloroacetic acid. The decalcified material was embedded in paraffin wax. From the resulting blocks, paraffin sections of 3–5 *μ*m thickness were cut and mounted on slides. The slides were stained with hematoxylin and eosin for light microscopy and polarized light analysis.

For each individual, we used a histological section from the T7-T8 and L4-L5 segments. The experimental groups designed to test our hypothesis were matched according to individual age and vertebral segment. There were a total of 41 specimens. Two groups were formed based on age: 20–40 years old (17 specimens) and 41–85 years old (24 specimens). In addition, two groups were formed according to the spinal segment: T7-T8 segments and L4-L5 segments.

**Figure 2:**
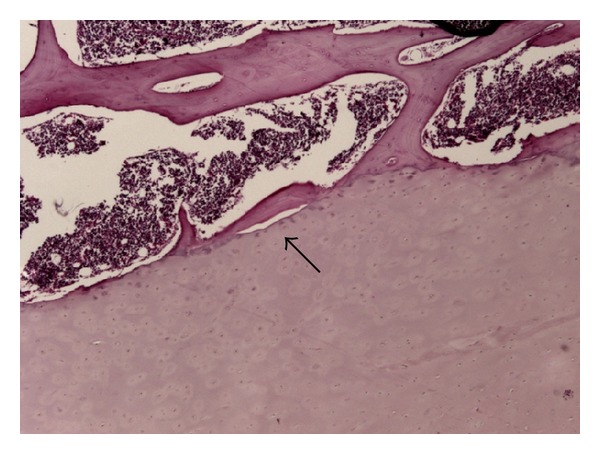
A separation (black arrow) was defined as the presence of space between the endplate and the cancellous bone of the vertebral body.

**Figure 3:**
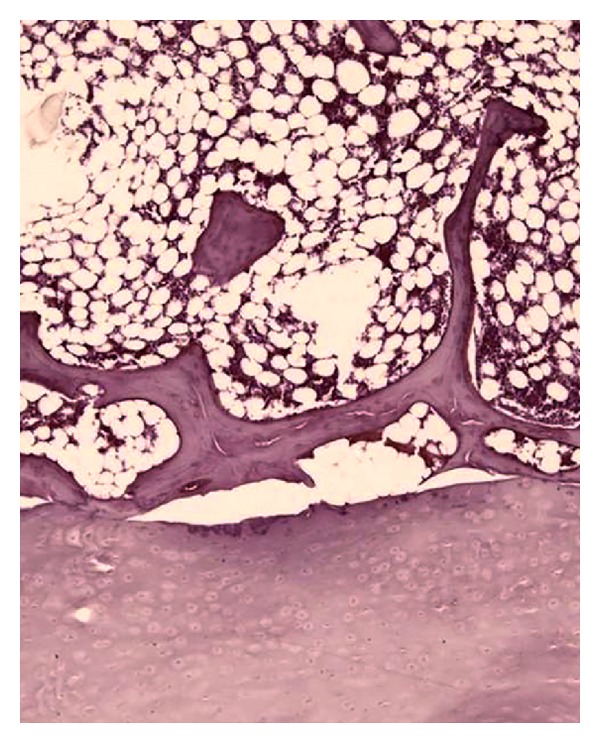
Separation suggesting an artifact occurred during preparation of the slides due to the presence of tears in the cancellous bone trabeculae.

The variables assessed by histological analysis were the length of the vertebral endplates and the number and dimensions of the separations between the cartilaginous endplate and the vertebral body. The length of the vertebra was the sum of the proximal and distal vertebral endplates. The endplate separations were defined as the presence of a space between the hyaline cartilage endplate and the perforated cortical bone of the adjacent vertebral body (Figure 2). Separations due to an artifact that occurred during preparation of the slides were considered when there was the presence of tears of the cancellous bone trabeculae and were excluded from evaluation (Figure 3). In order to differentiate vertebral endplate separations from possible artifacts, we also performed a qualitative analysis of the insertion of the collagen fibers from the osseous layers in the cartilaginous endplate. Collagen fibers were evaluated with polarized light microscopy, and the characteristic assessed was the regularity of the insertion of the fibers. To reduce the possible influence of the length of the vertebra on the study results, we created a separation index (SI) and a separation extension index (SEI). The SI was determined from the number of separations of the endplate divided by the length of the vertebra, and the SEI was calculated by the sum of all the separations of each segment divided by the length of the vertebra.

For statistical analysis, Student's *t*-test was used to evaluate the SI and to assess the statistical difference between the groups regarding the age (<40 years and >40 years) and the spinal level (thoracic and lumbar). The SEI was analyzed with the Kruskal-Wallis test and the groups were compared. A significance level of *P* < 0.05 was used in this study.

## 3. Results

**Figure 4:**
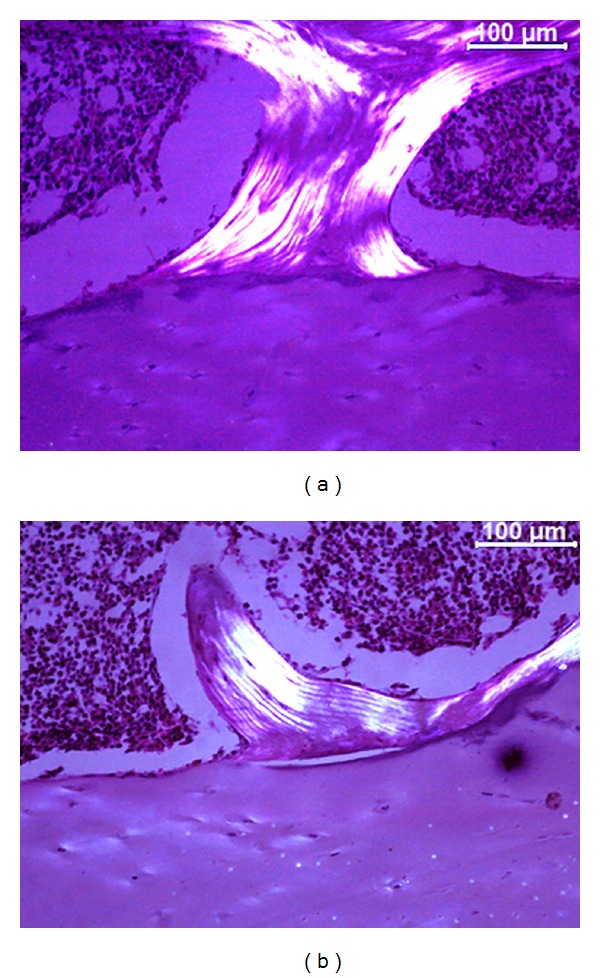
Polarized light microscopy images illustrating the collagen fiber insertion in a vertebral endplate area without separations (a) and in an area with separation (b).

Although separations between the vertebral body and the endplate have been demonstrated previously, there is a lack of evidence showing that the separations are not artifacts [13, 14]. To histologically differentiate between real separations of the vertebral endplate and artifacts is a difficult task; in our study, it was based on morphological features (trabecular fracture or tears) and the irregularity of collagen fiber insertions adjacent to the separation. Polarized light evaluation revealed that separation of the vertebral endplate presented irregular collagen fiber insertions, while more regular collagen insertion was observed in artifacts (Figure 4). This is demonstrated by the reduced lucent area in a vertebral endplate area without separations, when compared to the area adjacent to a vertebral endplate separation.

**Figure 5:**
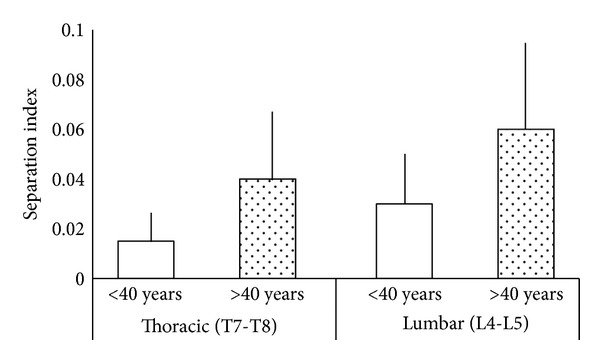
Comparison of the separation index (SI) in the thoracic (T7-T8) and lumbar (L4-L5) groups. Specimens from cadavers ≤40 years old and >40 years old were compared. Errors bars indicate the standard deviation.

**Figure 6:**
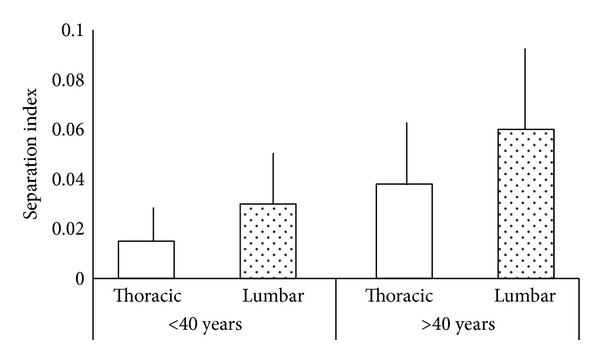
Comparison of the separation index (SI) in the ≤40 years and >40 years groups. Thoracic and lumbar segments were compared. Errors bars indicate the standard deviation.

There was no statistically significant difference in vertebral length (*P* > 0.05) between the group of individuals ≤40 years old and the group of individuals >40 years old, for either the thoracic segment or the lumbar segment. With increasing age, an increase in the SI was observed. The difference was significant for both segments: *P* = 0.0036 for the thoracic segment and *P* = 0.0038 for the lumbar segment (Figure 5). When the variable analyzed was the segment (thoracic and lumbar), the SI was significantly higher in the lumbar segment compared to the thoracic segment in both age groups (*P* = 0.0316 and 0.0193 for the younger and older groups, resp.) (Figure 6).

**Figure 7:**
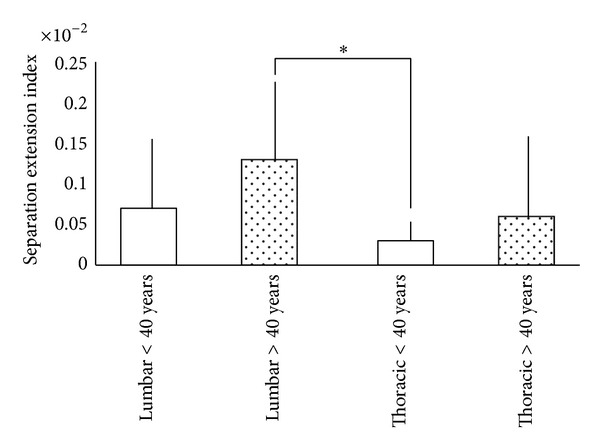
Comparison of the separation extension index (SEI) in the <40 years, >40 years, lumbar and thoracic groups. *Groups with statistical difference.

When the parameter evaluated was the separation extension index (SEI), there was a statistical difference between the lumbar >40 years group and the thoracic <40 years group (Figure 7) assessing the SEI (*P* = 0.003).

During microscopic evaluation, in certain specimens, we found fibrous formation within some separations, mostly in the >40 years group. Taken together, our findings indicate that separations are related to age and spinal level, and they suggest that the separations are not artifacts.

## 4. Discussion

The findings from this study demonstrate that separations between the endplate and the vertebral body depend on individual age and the spinal level. In our specimens, the index (SI) and extension (SEI) of the separations were both strongly associated with age and the spinal level. The few existing studies on vertebral endplate separations relied mainly on observations from a limited number of specimens per age group; moreover, they utilized only lumbar samples and did not mention the degenerative status of the intervertebral disc [12, 13, 15]. The majority of studies of vertebral endplates have addressed the transport properties of the vertebral endplate and its influence on disc degeneration [16–20]. The number of specimens in this study and our evaluation of both thoracic and lumbar segments allowed us to analyze a representative endplate length and look for associations with age and specific spinal level. To the best of our knowledge, this is the first time that endplate separations were quantified, measured, and analyzed with regard to age and spinal level in intervertebral discs without macroscopic or radiographic evidence of degeneration.

According to previous studies, the endplate may be more susceptible to mechanical failure than any other structure in the vertebral motion segment [4, 12]. Using finite element models, Natarajan et al. [4] demonstrated that mechanical failure of the intervertebral disc always began with separation of the endplate from the adjacent bone. In addition, microscopic observations identified clefts and tears that occurred in middle-aged patients but the intervertebral disc status was not taken into consideration [15]. This was confirmed by an autopsy study and it was postulated that the endplate could separate from the vertebral body [12]. Coventry et al. [15] identified these separations some time ago and observed several longitudinal fissures in the second and third decade of life. They considered the fissures to be artifacts, because the fissures did not, as a rule, show evidence of repair. However, as we found in our study, some specimens presented fibrous invasion inside the separations, suggesting that some separations are not artifacts. In addition, other studies have concluded there is a correlation between endplate separation and disc degeneration [12, 13].

Degenerative findings increase dramatically in specimens after the fourth decade of life [2, 15, 16, 18]. In our study, the association between the separation index and extension was strongest for the group over 40 years old and for the lumbar segment. Coventry et al. [15] also observed severe degenerative changes of the vertebral endplate in people 40 years old and older that ranged from thinning to complete absence of the cartilaginous endplate; however, only the lumbar segment was studied, and no distinctions between normal and degenerated discs were discussed. In older people, there were no specific changes in the cartilaginous vertebral endplate compared to the fourth decade of life, simply a progression of the previously noted degeneration [21, 22]. As the main conduit for nutrients and, potentially, a passage for metabolite removal, alterations in the vertebral endplate morphology can decrease or inhibit the transport process [7, 20, 23, 24]. Impaired nutrient and metabolite transport leads to a decrease in oxygen tension and glucose concentrations and an increase in lactate concentrations, causing cell viability alterations that affect matrix synthesis and lead to disc degeneration [9, 25–32]. Our results may correlate with biochemical and molecular alterations of the intervertebral disc. The most important causes of disc degeneration may be the various processes that weaken a disc before disruption or that impair its healing response. Further research is necessary to establish the relevance of vertebral endplate separations in the disc degeneration cascade.

This study has some limitations. The size of the vertebral body could interfere with the number of separations, so we decided to create the SI and the SEI; thus, evaluations of these parameters were relative to the size of the studied vertebrae. In addition, we are aware of the difficulty in differentiating between histological artifacts and separations of the vertebral endplate. Therefore, to decrease the chance of false-positive interpretations, the slides were assessed by polarized light microscopy in order to qualitatively evaluate the collagen fiber insertions of the vertebral endplate. The results of this analysis revealed that there were more irregularities in the separations between the vertebral endplate and the vertebral body than in artifacts. Another limitation of our study is the absence of magnetic resonance imaging in the radiological evaluation. The criteria used in our study to exclude severe degenerated intervertebral discs relied only on macroscopic and radiographic findings, but these techniques were able to demonstrate a statistical difference with aging. It is possible that including magnetic resonance imaging could provide more information than histological and radiographical findings alone.

## 5. Conclusion

Separations of the vertebral endplate, which are associated with age and spinal level, may be an aspect of the multifactorial nature of disc weakening that can lead to disc degeneration.
